# DEFINING OLD AGE IN SUB-SAHARAN AFRICA: THE PERSPECTIVE OF AFRICAN SCHOLARS AND PROFESSIONALS

**DOI:** 10.1093/geroni/igad104.2326

**Published:** 2023-12-21

**Authors:** Gifty Ashirifi, Abraham Teshome, Margaret Adamek, Dolapo Adeniji

**Affiliations:** Indiana University, Indianapolis, Indiana, United States; Indiana University, Indianapolis, Indiana, United States; Indiana University, Indianapolis, Indiana, United States; Indiana State University, Avon, Indiana, United States

## Abstract

The older adult population in Africa is projected to experience the fastest growth rate among regions in the world. Although in most Global North nations, 60-65 is considered old, the issue of what age should be accepted as “old” in Sub-Saharan Africa has remained a controversial issue with governments and scholars. The imminent increase in aging in Africa is a call for action since age definition is directly linked to access to programs and policies necessary to ensure older adults’ well-being. Through an online survey, this study investigated the perspectives of Gerontology and Social Work professionals from Sub-Saharan Africa (n=78, 55% female, age range 24-75) on what age should be accepted as “old” or “elderly” for policy development and research purposes. A descriptive analysis indicated that the most endorsed old age marker in Sub-Saharan Africa was 50 years old (33.3%) ---a full decade lower than the United Nation’s designation of old age. The primary factors respondents indicated should be considered when defining old age in Sub-Saharan Africa are health status (73.6%), retirement age (61.1%), and quality of life (56.9%). Age 60 was identified as the most appropriate age to determine eligibility for aging programs (45.1%) and for conducting research on later life (43.5%). We recommend that age guidelines used in Global South regions such as Sub-Saharan Africa should not necessarily mirror the guidelines for age used in the Global North but should fit the context of aging in those regions.

